# Supplementary material for: Systematic characterization of the local evolutionary space available to human PKR and vaccinia virus K3
Source: bioRxiv. 2023 Nov 22:2023.11.21.568178. Preprint. [Version 1] doi: 10.1101/2023.11.21.568178 (PMC10705557; doi:10.1101/2023.11.21.568178)
Supplement: Supplement 1 [file NIHPP2023.11.21.568178V1-supplement-1.pdf]

bioRxiv preprint doi: https://doi.org/10.1101/2023.11.21.568178; this version posted November 22, 2023. The copyright holder for this preprint (which was not certified by peer review) is the author/funder. This article is a US Government work. It is not subject to copyright under 17 USC 105 and is also made available for use under a CC0 license.

Supplementary figures

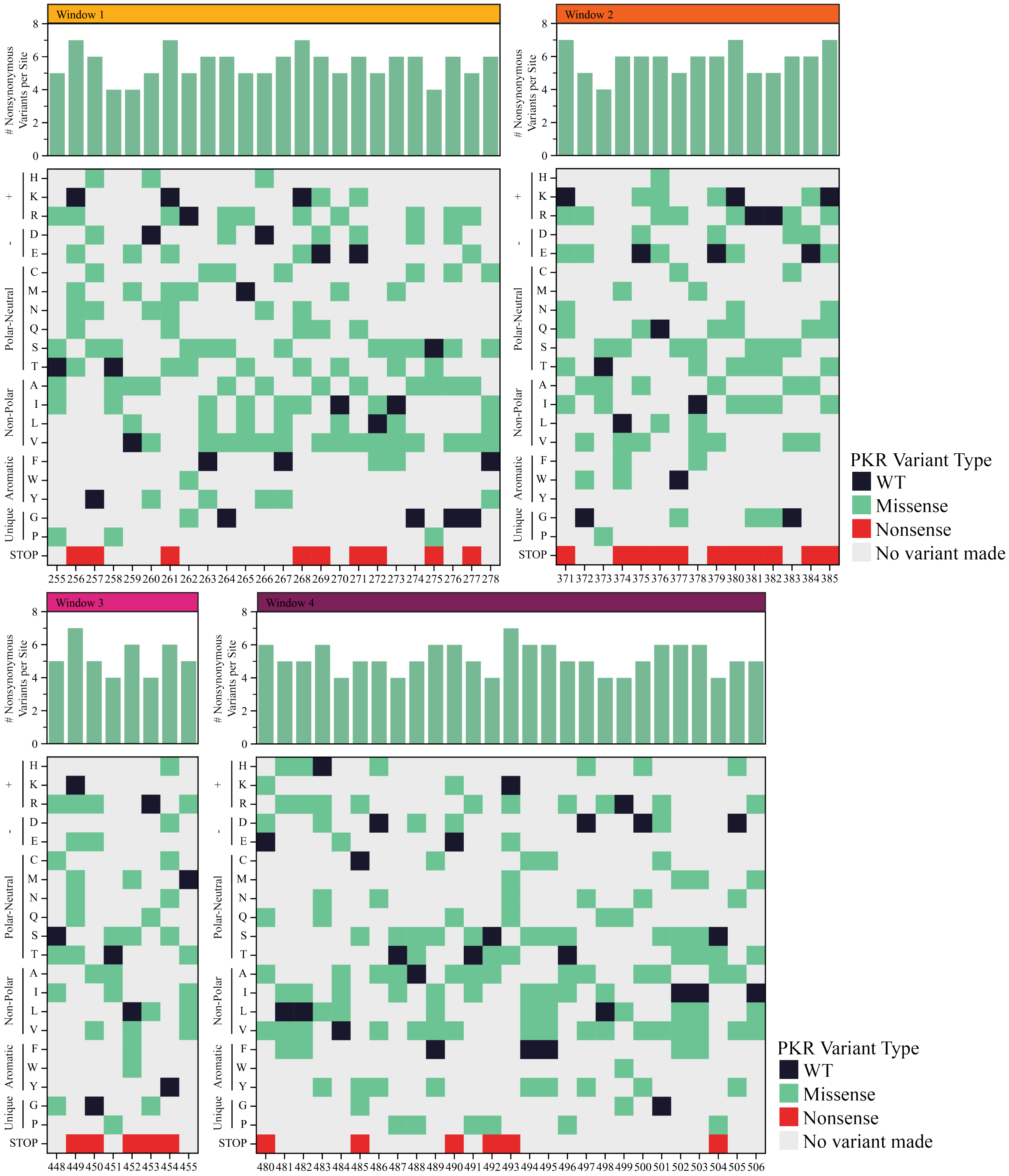

Figure S1. 409 nonsynonymous, SNP-accessible variants of interest were selected across four windows of interest in PKR. The top bar chart denotes the total number of variants that were made at each position, ranging from 4-8 variants. The sequence diagram highlights nonsynonymous, SNP-accessible *PKR* variants that were made across the four windows of interest in the PKR kinase domain, with missense variants in green, nonsense variants in red, and variants not made in gray. WT PKR residues are shown in black for reference.

bioRxiv preprint doi: https://doi.org/10.1101/2023.11.21.568178; this version posted November 22, 2023. The copyright holder for this preprint (which was not certified by peer review) is the author/funder. This article is a US Government work. It is not subject to copyright under 17 USC 105 and is also made available for use under a CC0 license.

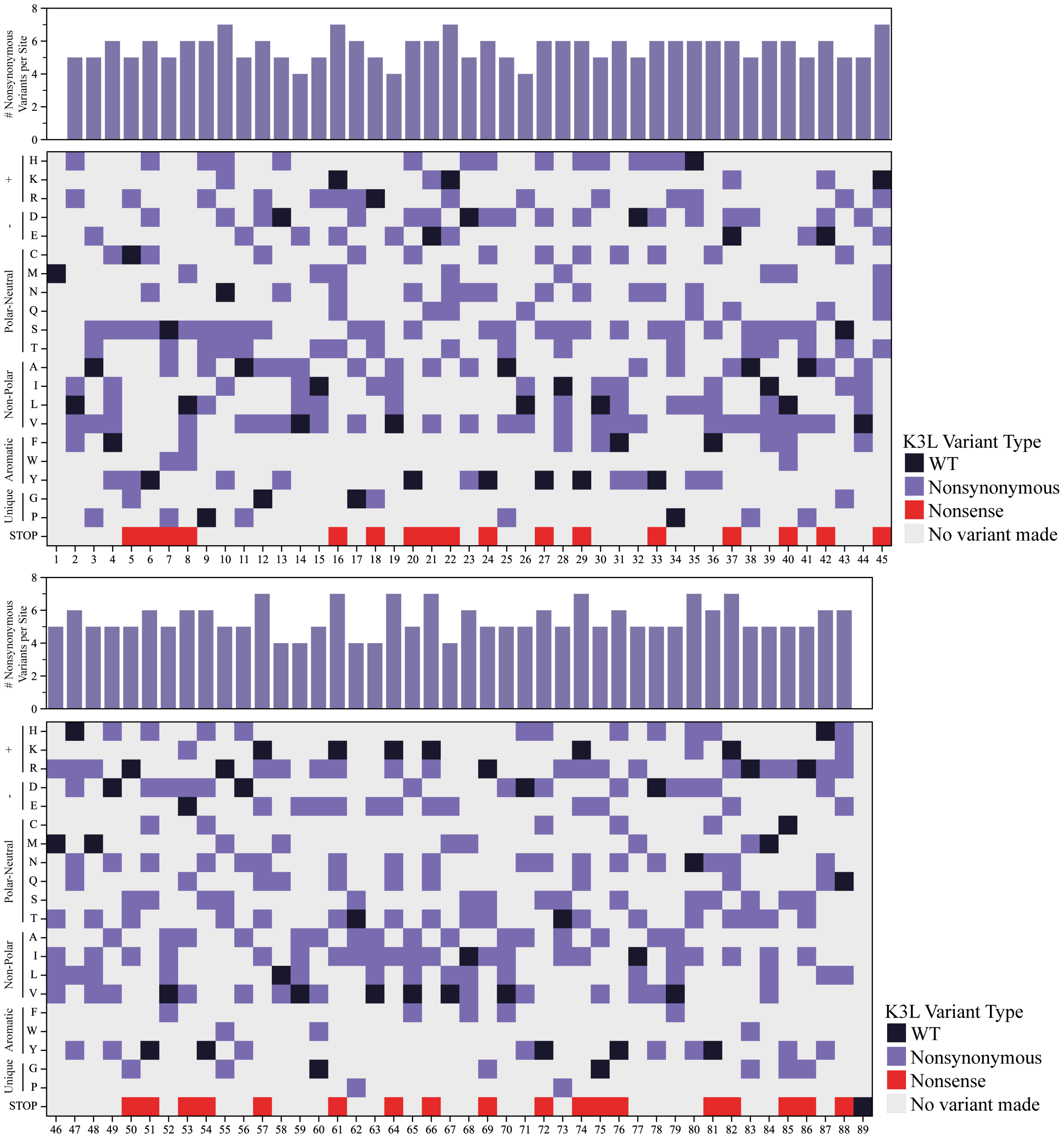

Figure S2. 500 nonsynonymous, SNP-accessible variants of interest were selected across the entirety of *K3L*. The top bar chart denotes the total number of variants that were made at each position, ranging from 5-8 variants. The sequence diagram highlights nonsynonymous, SNP-accessible *K3L* variants that were made across the entirety of K3 (L2-Q88), with missense variants in purple, nonsense variants in red, and variants not made in gray. WT K3 residues are shown in black for reference.

bioRxiv preprint doi: https://doi.org/10.1101/2023.11.21.568178; this version posted November 22, 2023. The copyright holder for this preprint (which was not certified by peer review) is the author/funder. This article is a US Government work. It is not subject to copyright under 17 USC 105 and is also made available for use under a CC0 license.

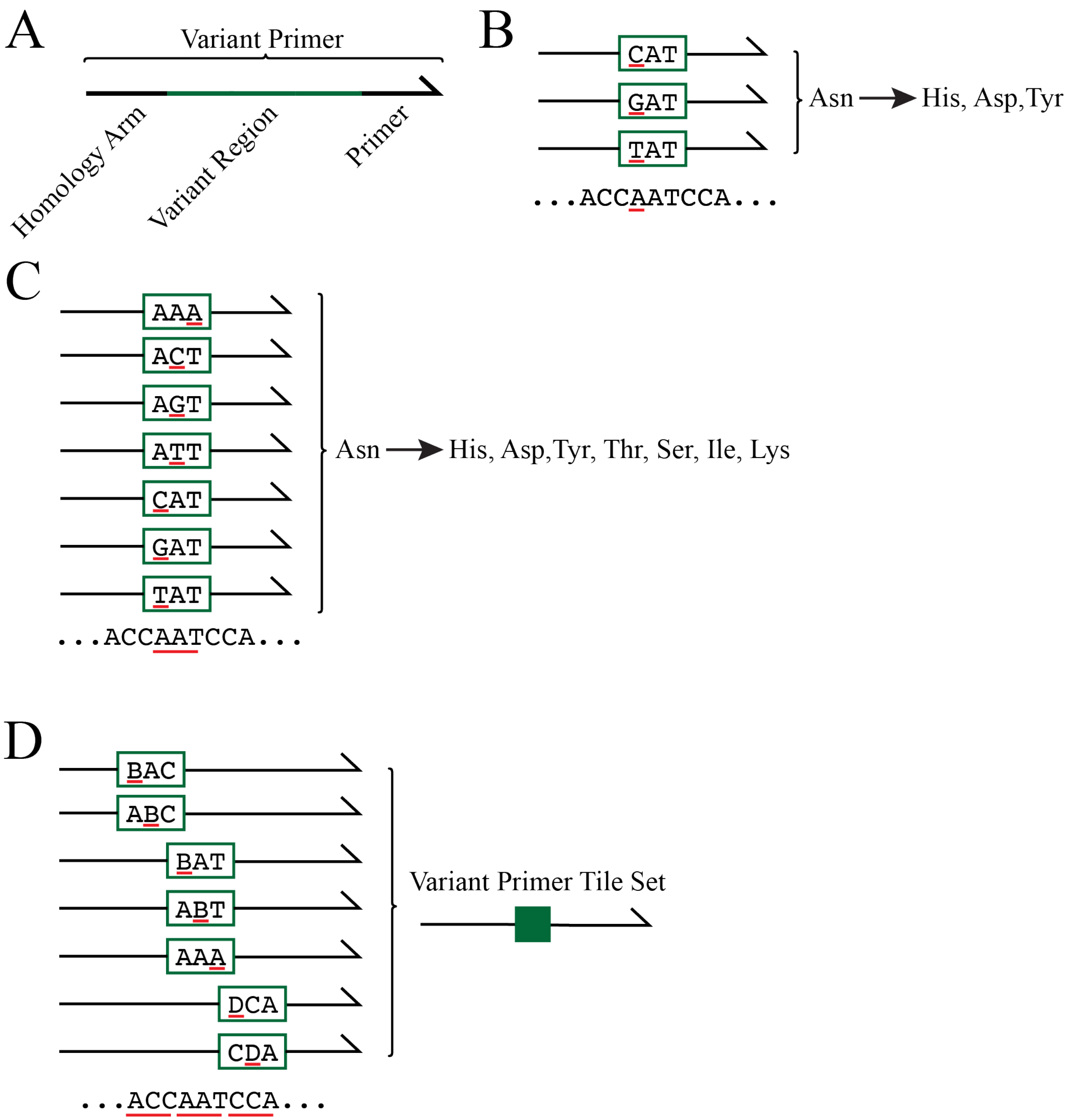

Figure S3. Variant primer tile sets were designed to systematically make single residue nonsynonymous variants. (A) Each variant primer is composed of a homology arm, variant region, and primer. **(B)** Nonsynonymous variants are generated by altering the codon in variant region of the primer. This example depicts the codon "AAT" encoding Asn. The first nucleotide in the codon, "A", is underlined in red, with three codons above having changes to "C", "G", and "T" underlined in red, which generate the nonsynonymous variants His, Asp, and Tyr. **(C)** Variant primers were designed across all three nucleotides in each codon, as underlined in red. **(D)** Variant primer tile sets, represented in dark green, were made by pooling variant primers that modify adjacent codons. Primers included in variant primer tile sets have differing variant regions but share homology arms and priming region sequences.

bioRxiv preprint doi: https://doi.org/10.1101/2023.11.21.568178; this version posted November 22, 2023. The copyright holder for this preprint (which was not certified by peer review) is the author/funder. This article is a US Government work. It is not subject to copyright under 17 USC 105 and is also made available for use under a CC0 license.

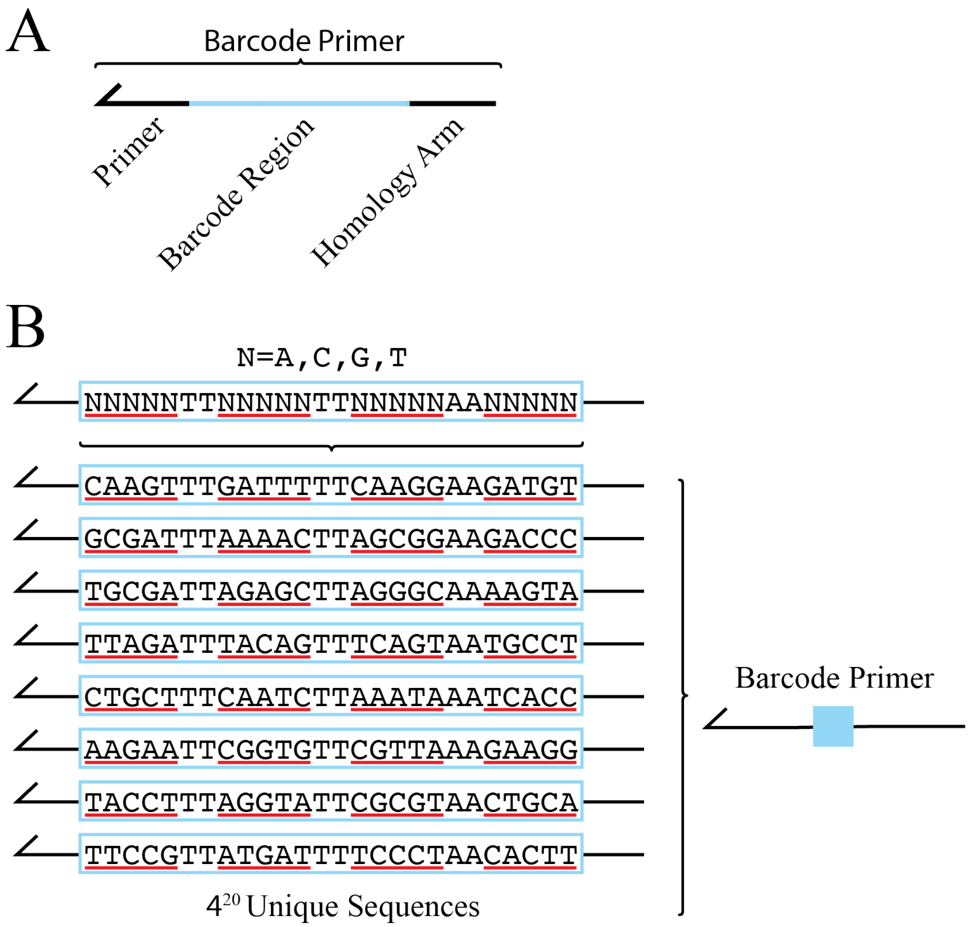

Figure S4. Barcode primers were designed to attach unique nucleotide sequences to each nonsynonymous variant. (A) The barcode primer is composed of a homology arm, barcode region, and primer. The barcode primer is paired with each of the variant primer tile sets to attach a unique nucleotide sequence to each nonsynonymous variant made. **(B)** The barcode region of the primer is composed of 20 "N", each "N" encodes an equal representation of the nucleotides "A", "C", "G", and "T". A barcode primer with 20 random nucleotides contains 420 unique nucleotide sequences. Dinucleotide sequences "TT" and "AA" are interspersed throughout the barcode region to avoid making unintended restriction enzyme cut sites.

bioRxiv preprint doi: https://doi.org/10.1101/2023.11.21.568178; this version posted November 22, 2023. The copyright holder for this preprint (which was not certified by peer review) is the author/funder. This article is a US Government work. It is not subject to copyright under 17 USC 105 and is also made available for use under a CC0 license.

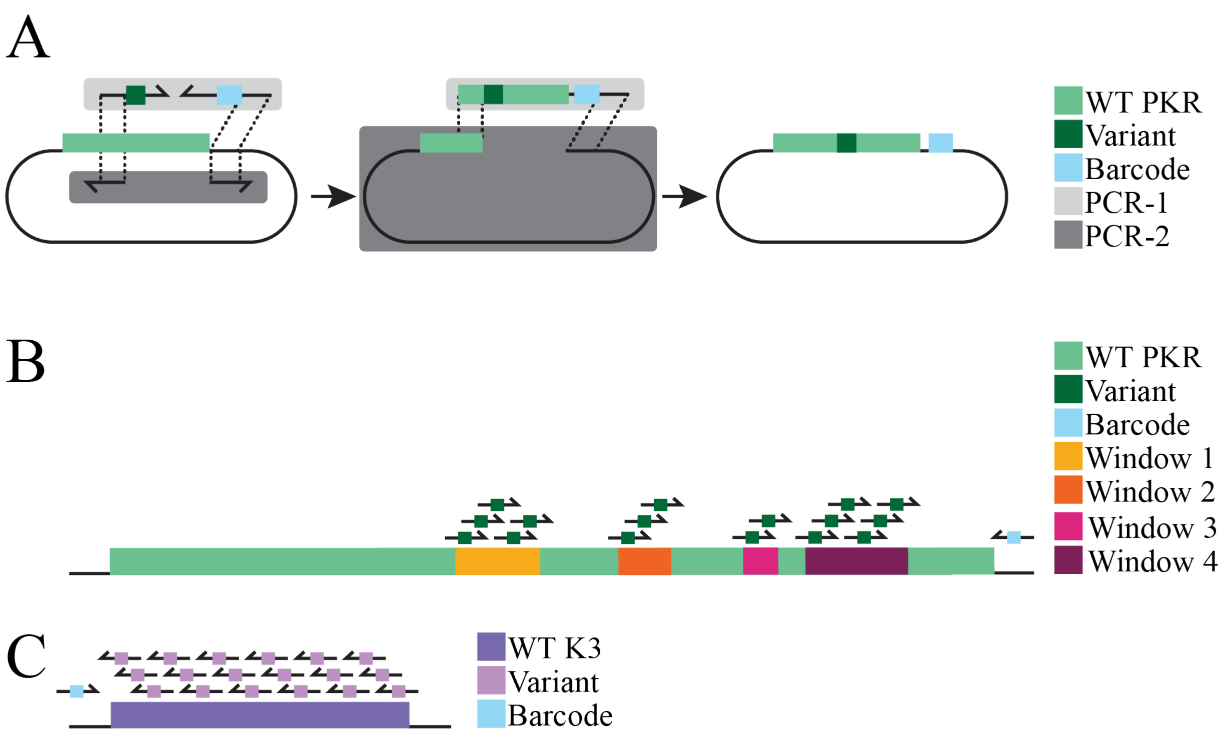

Figure S5. Single residue nonsynonymous variants were PCR amplified and assembled using doped primer tile sets across *PKR* and *K3L*. (A) Two separate PCR reactions were used to generate complementary insert and vector fragments. PCR-1 primers (light gray box) include a single variant primer tile set (dark green, see Figure S3D) and a single doped barcode primer (light blue, see Figure S4B) that amplified from WT *PKR* (green) and made the PCR-1 insert fragment containing a select nonsynonymous variant (dark green) and a unique barcode (blue). PCR-2 primers included a single forward and reverse primer that amplified from WT *PKR* and made larger vector fragment with 20 bp homology arms that complement the homology arms of the PCR-1 insert fragment. The two fragments were combined via Gibson Assembly to form a pool of complete vectors, each vector containing a single, nonsynonymous variant with a unique barcode. **(B)** 15 variant primer tile sets were designed to generate select variants across four windows of interest in the *PKR*. The full-length *PKR* sequence is denoted in green, with Windows 1-4 overlaid in yellow, orange, magenta, and burgundy. Variant primer tile sets encode select variants (dark green) and are paired with a single doped barcode primer (blue) for a total of 15 PCR-1 reactions for PKR that generate 435 variants. **(C)** 18 variant primer tile sets were designed to generate select variants across the entirety of *K3L*. The full length *K3L* sequence is denoted in purple; variant primer tile sets encode select variants (pink) and are paired with a single doped barcode primer (blue), making a total of 18 PCR-1 reactions for K3 that generate 527 variants.

bioRxiv preprint doi: https://doi.org/10.1101/2023.11.21.568178; this version posted November 22, 2023. The copyright holder for this preprint (which was not certified by peer review) is the author/funder. This article is a US Government work. It is not subject to copyright under 17 USC 105 and is also made available for use under a CC0 license.

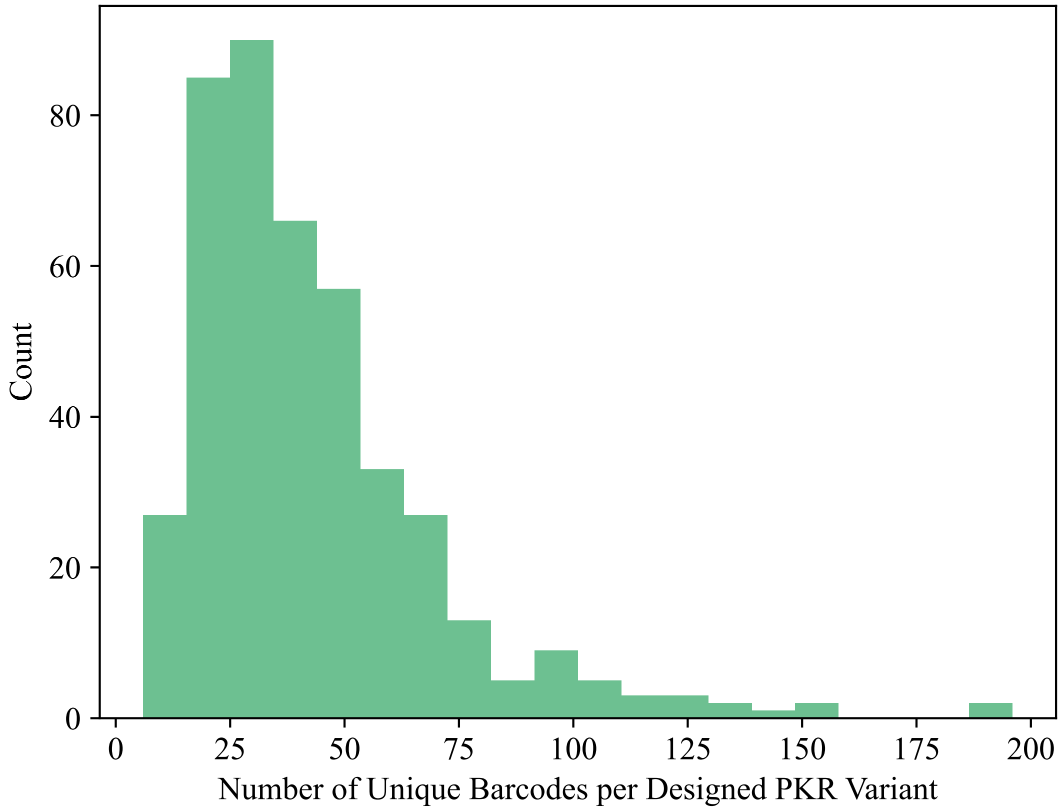

Figure S6. Count of unique barcodes per designed *PKR* variant. Histogram depicts the number of unique barcodes per designed *PKR* variant, with a mean of 43 barcodes per *PKR* variant.

bioRxiv preprint doi: https://doi.org/10.1101/2023.11.21.568178; this version posted November 22, 2023. The copyright holder for this preprint (which was not certified by peer review) is the author/funder. This article is a US Government work. It is not subject to copyright under 17 USC 105 and is also made available for use under a CC0 license.

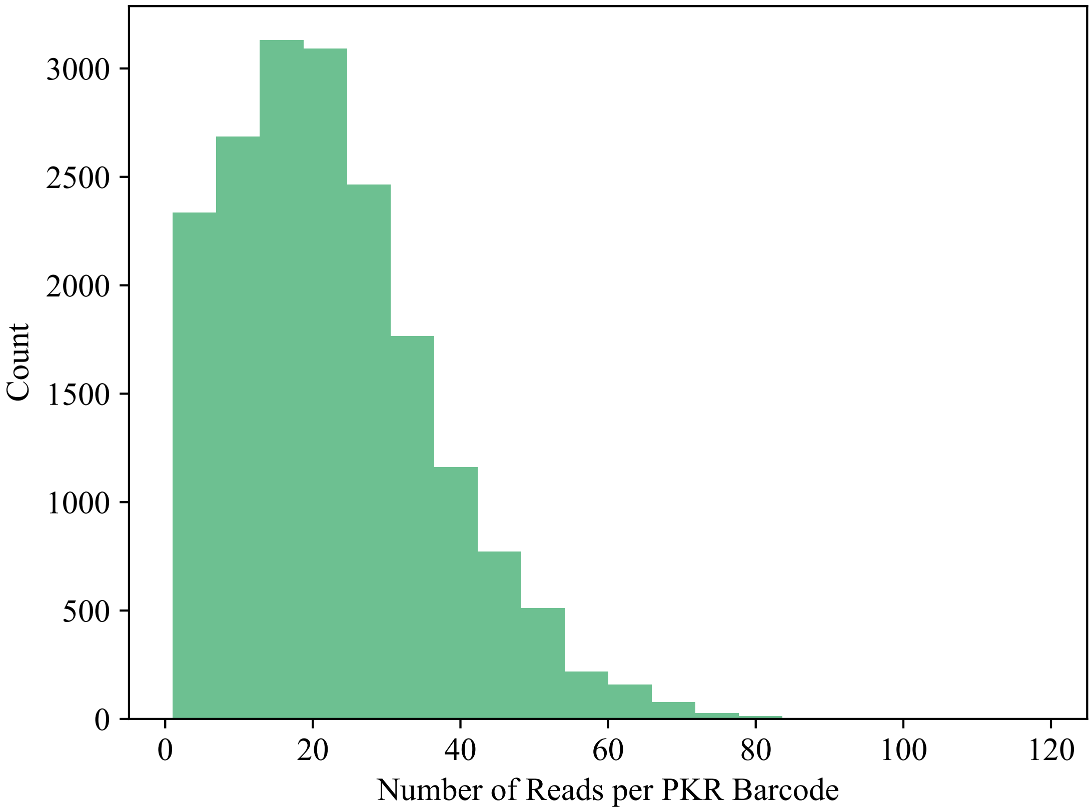

Figure S7. Count of unique barcodes per designed *PKR* variant. Histogram depicts the number of unique barcodes per designed *PKR* variant, with a mean of 43 barcodes per *PKR* variant.

bioRxiv preprint doi: https://doi.org/10.1101/2023.11.21.568178; this version posted November 22, 2023. The copyright holder for this preprint (which was not certified by peer review) is the author/funder. This article is a US Government work. It is not subject to copyright under 17 USC 105 and is also made available for use under a CC0 license.

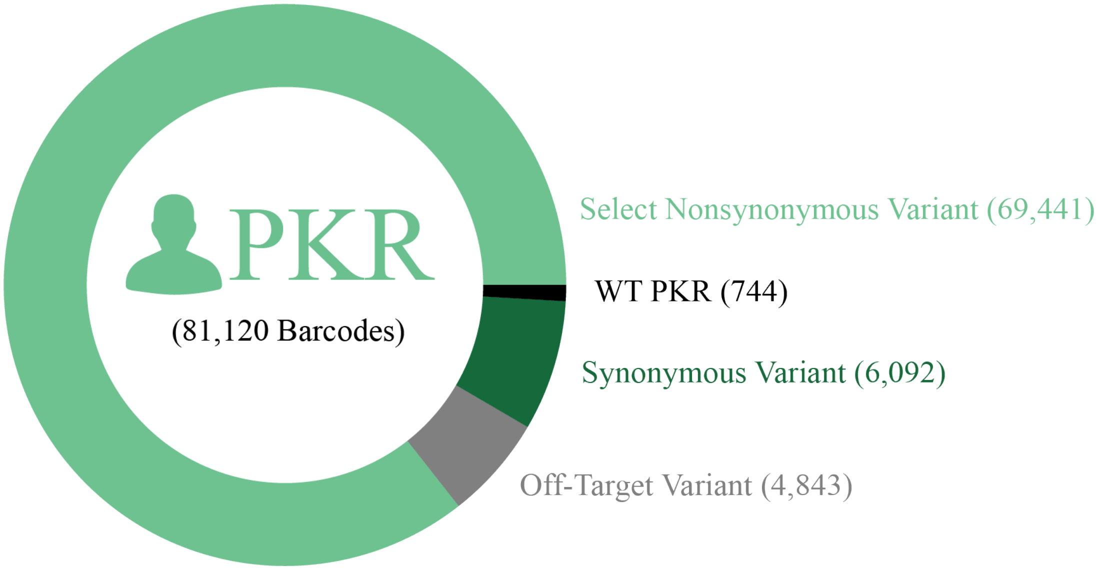

Figure S8. PacBio barcode distribution linked to *PKR* variant types. A total of 81,120 barcodes were identified in the *PKR* variant library. 86% of the barcodes were linked to select nonsynonymous variants of PKR (69,441 barcodes), 8% of the barcodes linked to either WT PKR or synonymous variants (744 and 6,092 barcodes, respectively), and 6% of the barcodes linked to off-target variants (4,843 barcodes). Off-target variants are barcodes linked to nonsynonymous variants outside the windows of interest or contain >1 nonsynonymous variant.

bioRxiv preprint doi: https://doi.org/10.1101/2023.11.21.568178; this version posted November 22, 2023. The copyright holder for this preprint (which was not certified by peer review) is the author/funder. This article is a US Government work. It is not subject to copyright under 17 USC 105 and is also made available for use under a CC0 license.

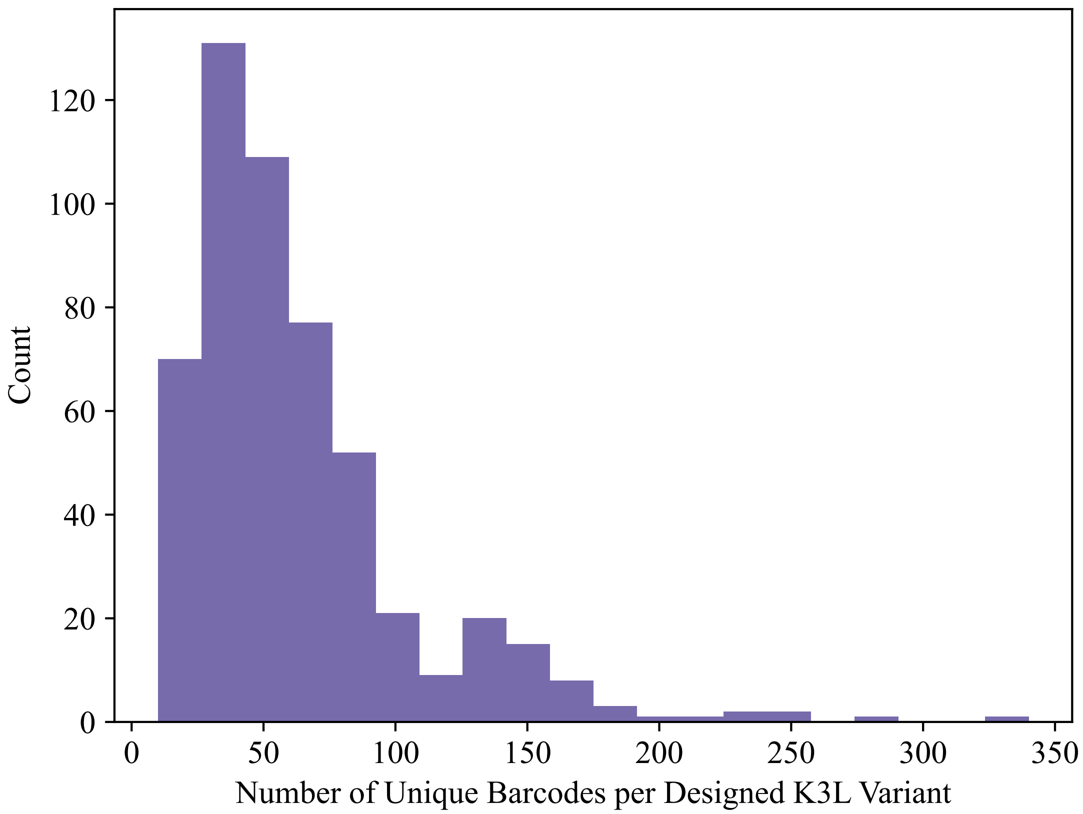

Figure S9. Count of unique barcodes per designed *K3L* variant. Histogram depicts the number of unique barcodes per designed *K3L* variant with a mean of 63 barcodes per *K3L* variant.

bioRxiv preprint doi: https://doi.org/10.1101/2023.11.21.568178; this version posted November 22, 2023. The copyright holder for this preprint (which was not certified by peer review) is the author/funder. This article is a US Government work. It is not subject to copyright under 17 USC 105 and is also made available for use under a CC0 license.

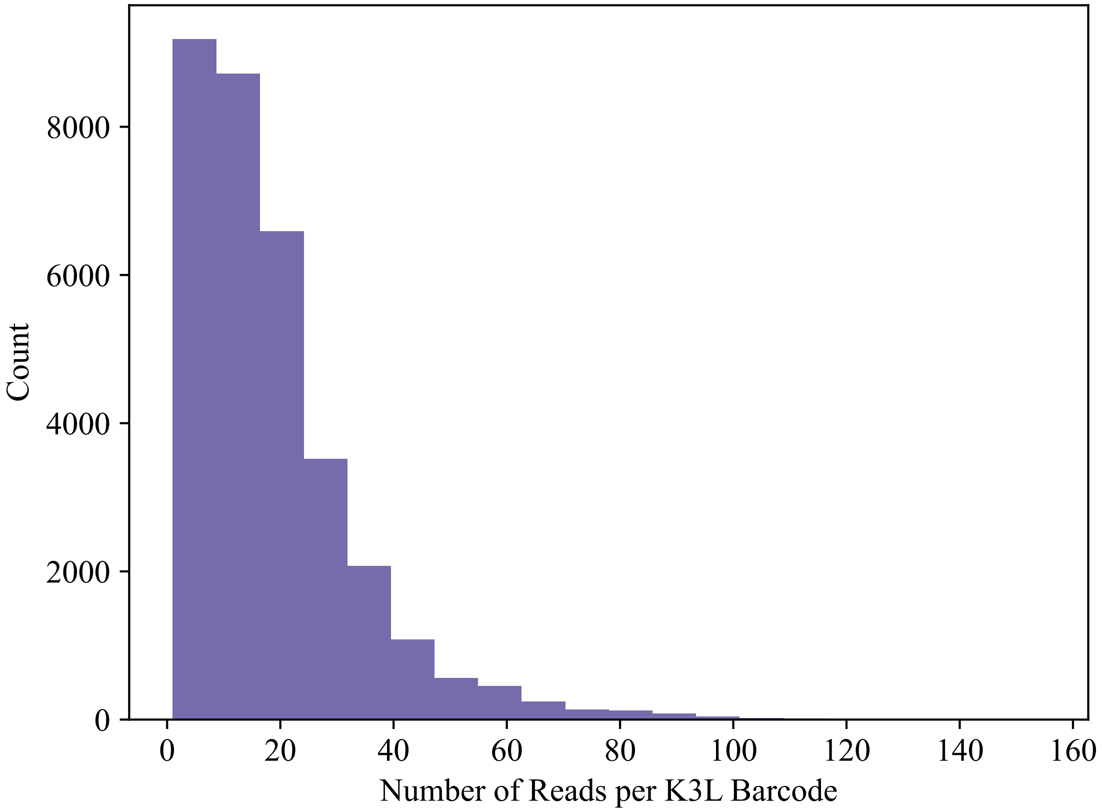

Figure S10. PacBio read depth per *K3L* barcode. Histogram depicts the number of reads per *K3L* barcode with a mean of 18 reads per barcode.

bioRxiv preprint doi: https://doi.org/10.1101/2023.11.21.568178; this version posted November 22, 2023. The copyright holder for this preprint (which was not certified by peer review) is the author/funder. This article is a US Government work. It is not subject to copyright under 17 USC 105 and is also made available for use under a CC0 license.

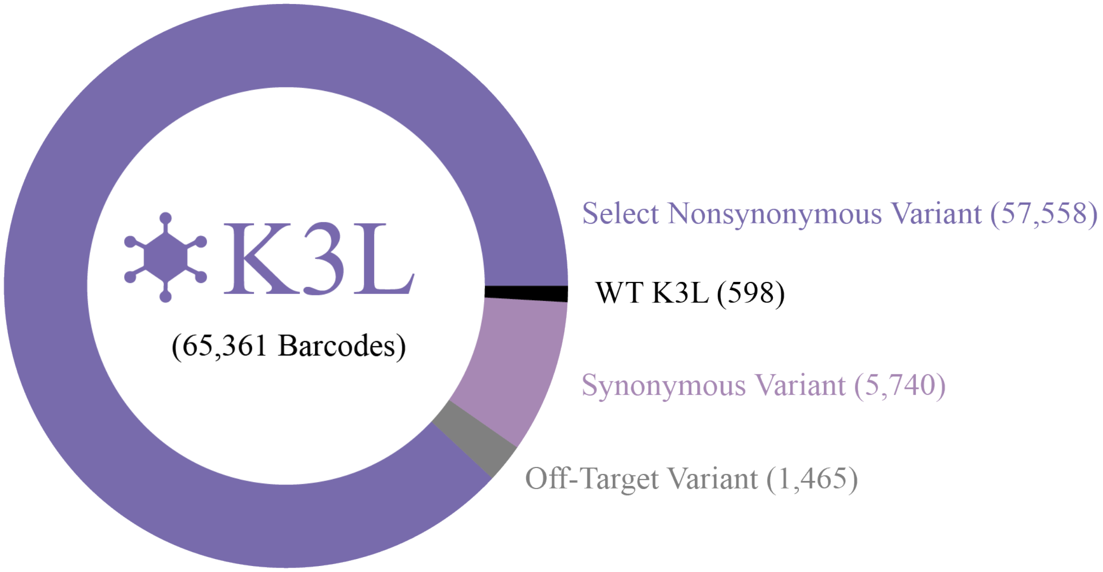

Figure S11. PacBio barcode distribution linked to *K3L* variant types. A total of 65,361 barcodes were identified in the *K3L* variant library. 88% of the barcodes were linked to select nonsynonymous variants of *K3L* (57,558 barcodes), 10% of the barcodes were linked to either WT K3 or synonymous variants (598 and 5740 barcodes, respectively), and 2% of the barcodes were linked to off-target variants (1,465 barcodes). Off-target variants are barcodes linked to nonsynonymous variants outside residues L2-Q88 or contain >1 nonsynonymous variant.

bioRxiv preprint doi: https://doi.org/10.1101/2023.11.21.568178; this version posted November 22, 2023. The copyright holder for this preprint (which was not certified by peer review) is the author/funder. This article is a US Government work. It is not subject to copyright under 17 USC 105 and is also made available for use under a CC0 license.

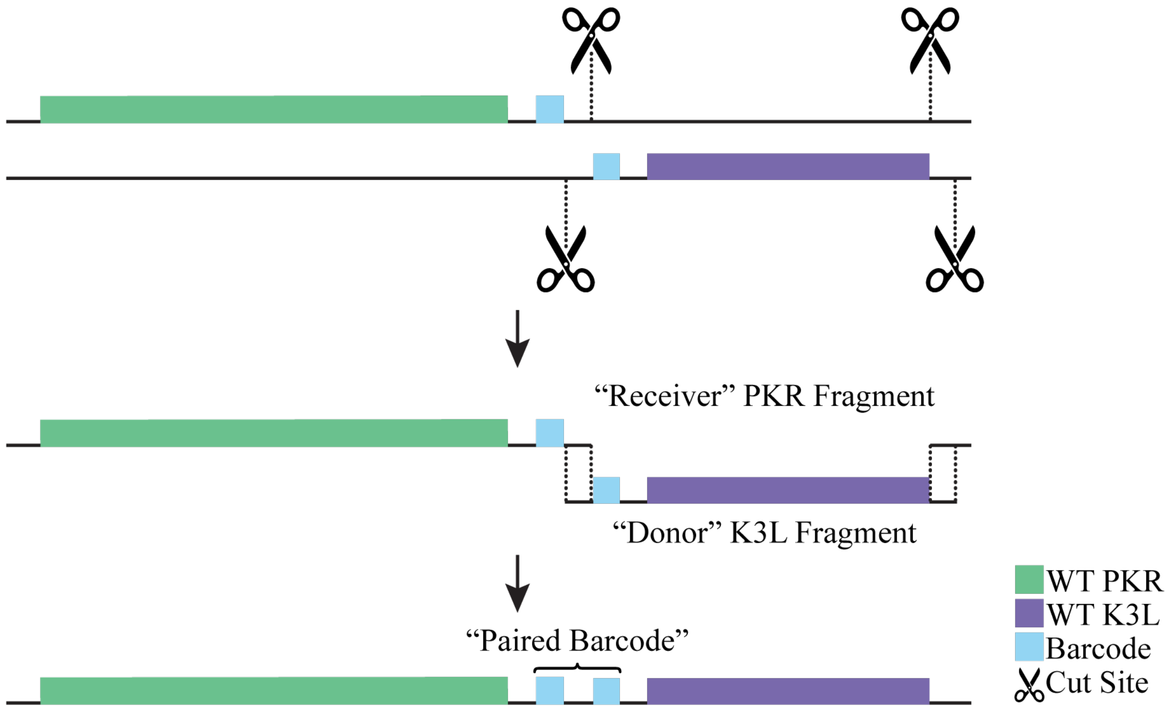

Figure S12. *PKR* and *K3L* variant libraries were combined to form an all-pairs library containing all 204,500 unique pairs of *PKR* and *K3L* variants with adjacent barcodes to form a "paired barcode". A double-digest of the *PKR* variant library creates a landing pad with homology to a double-digest fragment from the *K3L* variant library using unique restriction enzyme cut sites for each variant library. The *PKR* and *K3L* fragments are flanked with 20 bp homology arms that complement one another for Gibson Assembly, forming a library of 204,500 unique pairs. *PKR* and *K3L* barcodes are placed adjacent to one-another to form a "paired barcode" that can be read via Illumina short-read sequencing.

bioRxiv preprint doi: https://doi.org/10.1101/2023.11.21.568178; this version posted November 22, 2023. The copyright holder for this preprint (which was not certified by peer review) is the author/funder. This article is a US Government work. It is not subject to copyright under 17 USC 105 and is also made available for use under a CC0 license.

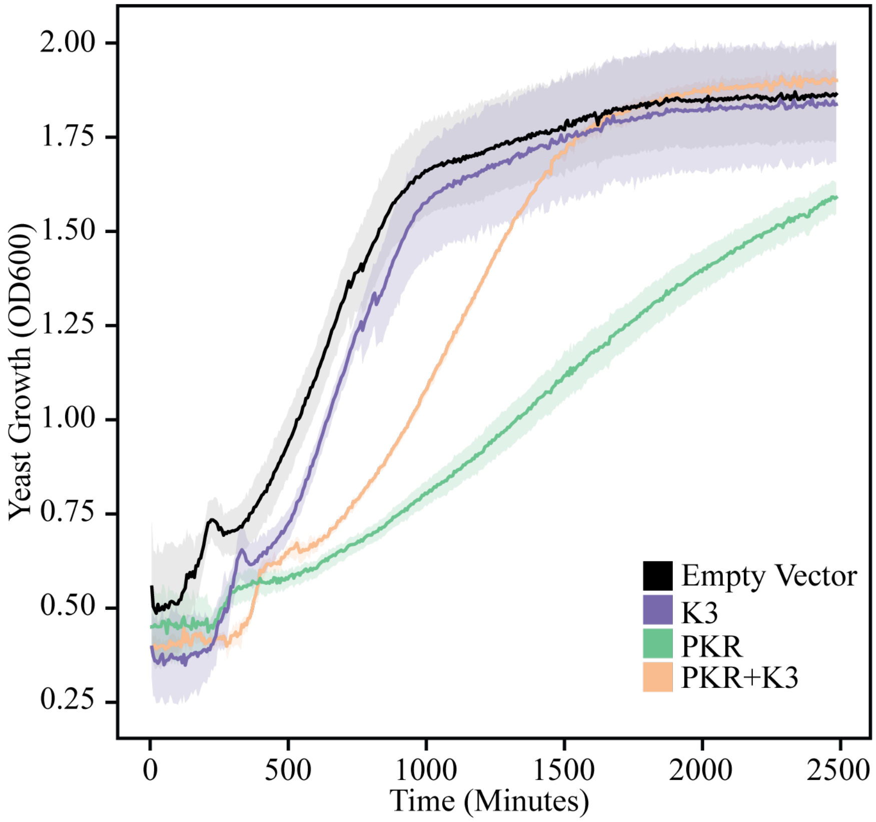

Figure S13. Expression of PKR and K3 alters yeast growth rates. Expression of PKR, K3, or a combination of the two in yeast alters growth over time. WT yeast growth (black) is comparable to yeast expressing K3 (purple), while yeast growth is inhibited when expressing PKR (green). However, yeast growth is appreciably recovered when both PKR and K3 are co-expressed (orange), as K3 antagonizes PKR inhibition of translation and growth.

bioRxiv preprint doi: https://doi.org/10.1101/2023.11.21.568178; this version posted November 22, 2023. The copyright holder for this preprint (which was not certified by peer review) is the author/funder. This article is a US Government work. It is not subject to copyright under 17 USC 105 and is also made available for use under a CC0 license.

Supplementary tables

Table S1 Oligos used to generate and characterize *PKR* and *K3L* variants.

Oligo#	Sequence
Oligo 1	cctttcgtcttcaaGAATTCgcatctACCGGTtaagcgCGTACGtgcatgGGTGACCaagtttctgtacaacaatcaGCGGCCGCaacgtcACGCGTgtactcCAGCTGctacagatccgcataagGAGCTCatcgataagctttaatgcggtagttta
Oligo 2	GTCTTCAAGAATTCGCATCTACCGGTATAACCATAAAAGCTAGTATTGTAGAATCTTTATTG
Oligo 3	CAGAGAAAAATGAACGACACACATGTTAGTAGCGAAAGCTCACTGACCGTATATATATATAACTGTCTAGAAATAAAGAGTATCATCTTTCAAATCAAGCTCTGCGAGTATGCTAACCAAGGCGCTTAATATGCAATGAAATTGCTTAGGTGACCAAGTTTCTGTACAACAATCA
Oligo 4	CAGAGAAAAATGAACGACACACATGTTAGTAGCGAAAGCTCACTGACCGTATATATATATAACTGTCTAGAAATAAAGAGTATCATCTTTCAAATCAAGCTCTGCGAGTATGCTAACCAAGGCGCTTAATATGCAATGAAATTGCTTAGGTGACCAAGTTTCTGTACAACAATCA
Oligo 5	GATTTCATACACGGTGCCTGAC
Oligo 6	AAGTTTCTGTACAACAATCAGCGGCCGCAGTTAAATCGTTTTCGAGATTTTCGCGACAGACCCATCTGTATGCTCGGCTAACTTTGAAAGATGATACTCTTTATTCCTACATAAGTAAATGAGTTTATATAGAGCTGCGACTCGACAGCTACTATTGATGTCTACACATCCTTTTGTAATTGACATC
Oligo 7	AACACACATAAACAAACAAAACGCGTCCATGCTTGCATTTTGTTATTCGTTGCCCAATG
Oligo 8	TTTGTTTGTTTATGTGTGTTTATTCGAAACTAAG
Oligo 9	CTCCTTATGCGGATCTGTAGCAGCTGTCATTATCAATACTGCCATTTCAAAGAATAC
Oligo 10	PKR variant primer tile set 1 fwd
Oligo 11	PKR variant primer tile set 2 fwd
Oligo 12	PKR variant primer tile set 3 fwd
Oligo 13	PKR variant primer tile set 4 fwd
Oligo 14	PKR variant primer tile set 5 fwd
Oligo 15	PKR variant primer tile set 6 fwd
Oligo 16	PKR variant primer tile set 7 fwd
Oligo 17	PKR variant primer tile set 8 fwd
Oligo 18	PKR variant primer tile set 9 fwd
Oligo 19	PKR variant primer tile set 10 fwd
Oligo 20	PKR variant primer tile set 11 fwd
Oligo 21	PKR variant primer tile set 12 fwd
Oligo 22	PKR variant primer tile set 13 fwd
Oligo 23	PKR variant primer tile set 14 fwd
Oligo 24	PKR variant primer tile set 15 fwd

bioRxiv preprint doi: https://doi.org/10.1101/2023.11.21.568178; this version posted November 22, 2023. The copyright holder for this preprint (which was not certified by peer review) is the author/funder. This article is a US Government work. It is not subject to copyright under 17 USC 105 and is also made available for use under a CC0 license.

Table S1 continued

Oligo#	Sequence
Oligo 25	K3L variant primer tile set 1 fwd
Oligo 26	K3L variant primer tile set 2 fwd
Oligo 27	K3L variant primer tile set 3 fwd
Oligo 28	K3L variant primer tile set 4 fwd
Oligo 29	K3L variant primer tile set 5 fwd
Oligo 30	K3L variant primer tile set 6 fwd
Oligo 31	K3L variant primer tile set 7 fwd
Oligo 32	K3L variant primer tile set 8 fwd
Oligo 33	K3L variant primer tile set 9 fwd
Oligo 34	K3L variant primer tile set 10 fwd
Oligo 35	K3L variant primer tile set 11 fwd
Oligo 36	K3L variant primer tile set 12 fwd
Oligo 37	K3L variant primer tile set 13 fwd
Oligo 38	K3L variant primer tile set 14 fwd
Oligo 39	K3L variant primer tile set 15 fwd
Oligo 40	K3L variant primer tile set 16 fwd
Oligo 41	K3L variant primer tile set 17 fwd
Oligo 42	K3L variant primer tile set 18 fwd
Oligo 43	TGATTGTTGTACAGAAACTTGGTCACCNNNNNAANNNNNTTNNNNNTTNNNNNCCTTGGTTAGCATACTCGCAGA
Oligo 44	AAGTTTCTGTACAACAATCAGCGGCCGCNNNNNAANNNNNTTNNNNNTTNNNNNGACAGACCCATCTGTATGCTCG
Oligo 45	AAGTTTCTGTACAACAATCAGCGG
Oligo 46	TGATTGTTGTACAGAAACTTGGTCAC
Oligo 47	TTCTTTCATGTCAGGAAGGTCAAATCTG
Oligo 48	GTCCACAGTATACTTTGTTTCTTTCATGTC
Oligo 49	GCCAAACCTCTTGTCCACAGTATAC
Oligo 50	TATTTCTTTAAAATCCATGCCAAACCTCTTG
Oligo 51	ACCTGAGCCAATTAATTCTATTTCTTTAAAATCC
Oligo 52	ATCACAGAATTCCATTTGGATGAAAAGGC
Oligo 53	TTGTTCCAAGGTCCCTTTATCACAGA
Oligo 54	TCTTCTTTTTTCAATCCATTGTTCCAAGG
Oligo 55	CCTTGTTCGCTTTCCATCATTTTTCA
Oligo 56	TCGCAAAGTTCCCTTACTCCTTGT
Oligo 57	AGCAAGAATTAGCCCCAAAGCG
Oligo 58	ACATACATGAAGAAGTTCAGCAAGAATTAGC
Oligo 59	TTCAAAAGCAGTGTCACATACATGAAGA
Oligo 60	GTCTGTGAAAAACTTTGATGTTTCAAAAGC
Oligo 61	GCCATCCCGTAGGTCTGTGA
Oligo 62	CATGGACGCGTTTTGTTTGTTTATGT
Oligo 63	CAACGAATAACAAAATGCAAGCATGG
Oligo 64	TACATCACCCGCATTGGGCA

bioRxiv preprint doi: https://doi.org/10.1101/2023.11.21.568178; this version posted November 22, 2023. The copyright holder for this preprint (which was not certified by peer review) is the author/funder. This article is a US Government work. It is not subject to copyright under 17 USC 105 and is also made available for use under a CC0 license.

Table S1 continued

Oligo#	Sequence
Oligo 65	TCTGCCCTTTATTACATCACCCGC
Oligo 66	CTTCTCGTATACTCTGCCCTTTATTACATC
Oligo 67	TAGAGCATAATCCTTCTCGTATACTCTGC
Oligo 68	AAAAAGATAAATATATAGAGCATAATCCTTCTCGTATACT
Oligo 69	TTCAAAGTGAGGATAGTCAAAAAGATAAATATATAGAGC
Oligo 70	CTCTGCCAAGATAGCTTCAAAGTGA
Oligo 71	CATCTTAACACTCTCTGCCAAGATAGC
Oligo 72	ATATCTATCCATATGCATCTTAACACTCTCTGC
Oligo 73	TTTATCCCTATATTCAACATATCTATCCATATGCATC
Oligo 74	TTTCCCTACCAGTTTATCCCTATATTCAACA
Oligo 75	AACTTTTACAGTTTTCCCTACCAGTTTATCC
Oligo 76	TCTAATCACTTTAACTTTTACAGTTTTCCCTACC
Oligo 77	TGTATAATCAACTCTAATCACTTTAACTTTTACAGTTTT
Oligo 78	ATCTATATATCCTTTTGTATAATCAACTCTAATCACTTTAACT
Oligo 79	CCTTTTGTAATTGACATCTATATATCCTTTTGTATAATCA
Oligo 80	TCGTCGGCAGCGTCAGATGTGTATAAGAGACAGTCAAGCTCTGCGAG TATGCTAAC
Oligo 81	TCGTCGGCAGCGTCAGATGTGTATAAGAGACAGNTCAAGCTCTGCGA GTATGCTAAC
Oligo 82	TCGTCGGCAGCGTCAGATGTGTATAAGAGACAGNNTCAAGCTCTGCG AGTATGCTAAC
Oligo 83	TCGTCGGCAGCGTCAGATGTGTATAAGAGACAGNNNTCAAGCTCTGC GAGTATGCTAAC
Oligo 84	TCGTCGGCAGCGTCAGATGTGTATAAGAGACAGNNNNTCAAGCTCTG CGAGTATGCTAAC
Oligo 85	GTCTCGTGGGCTCGGAGATGTGTATAAGAGACAGGTTAGCCGAGCAT ACAGATGGG
Oligo 86	GTCTCGTGGGCTCGGAGATGTGTATAAGAGACAGNGTTAGCCGAGCA TACAGATGGG
Oligo 87	GTCTCGTGGGCTCGGAGATGTGTATAAGAGACAGNNGTTAGCCGAGC ATACAGATGGG
Oligo 88	GTCTCGTGGGCTCGGAGATGTGTATAAGAGACAGNNNGTTAGCCGAG CATACAGATGGG
Oligo 89	GTCTCGTGGGCTCGGAGATGTGTATAAGAGACAGNNNNGTTAGCCGA GCATACAGATGGG
Oligo 90	Paired barcode fwd, pooled Oligos 80-84
Oligo 91	Paired barcode rev, pooled Oligos 85-89
